# Supplementary material for: The magnitude of undernutrition and associated factors among adult chronic kidney disease patients in selected hospitals of Addis Ababa, Ethiopia
Source: PLoS One. 2021 Jul 8;16(7):e0251730. doi: 10.1371/journal.pone.0251730 (PMC8266056; doi:10.1371/journal.pone.0251730)
Supplement: S1 File — (DOCX) [file pone.0251730.s001.docx]

## S1 File: Analytical procedures done for Albumin determination

| Principle | Human albumin forms a precipitate with a specific antiserum which is determined turbid metrically at 340 nm.  **Materials**   \| **Reagents** \| \| --- \| \| 1. ALB Cassettes Catalog No. 04469658 190  2. NaCl Diluent 9 % Catalog No. 20756350 322  3. Deproteinizer Catalog No.20 763 071 122  4. Cleaner Catalog No.20 754 765 322 \|   **Reagents preparation**: Ready for use.  **Reagents stability and storage**:  Shelf life at 2‑8 °C See expiration date on cobas c pack label  COBASINTEGRA 400 plus systems  On-board in use at 10 to15°C 12 weeks   \| **Supplies** \| \| --- \| \| 1. Sample Cup micro 0.5ml Catalog 11 406 680 001 \| \| 1. NaCl Diluent 9 % Cat. No. 20756350 322 \| \| 3. Waste Container Catalog 21 044 850 001 \| \| 4. Cleaner Cassette Catalog 20 764 337 322 \| \| 5. Micro Cuvettes Catalog 21 043 862 001 \| \| **Equipment** \| \| - CobasIntegra 400 & 501 plus analyzer • Centrifuge - Micro pipette( 20-1000µl) • Sample rack - Vortex \|   **Sample**   \| Sample type \| Amount required \| Transport and Storage \| Stability \| \| --- \| --- \| --- \| --- \| \| Serum/Plasma: Li-heparin, K2- or K3-EDTA \| 0.3mL \| -Transport whole blood at RT -Separate serum within 1 Hr.  - Store serum at 2-8⁰C or -20⁰C \| 10 days at 15-25°C  5 months at 2-8 °C  4 months at (‑15)‑(‑25) °C \| |
| --- | --- | --- | --- | --- | --- | --- | --- | --- | --- | --- | --- | --- | --- | --- | --- | --- | --- | --- | --- |

*Note: -* Freeze only once.

**Limitations**: Gross hemolysis, lipemic and icterus specimen.

**Sample retention**: Specimens are discarded in accordance with EPHI Specimen retention policy. This refers to both Specimens in the Primary and Secondary Containers.

| **Result Interpretation**   \| Step \| Action \| \| --- \| --- \| \| 1 \| Lower detection limit of the test: 3 g/L (46 μmol/L or 0.3 g/dL)  The lower detection limit represents the lowest measurable analyte level that can be distinguished from zero. It is calculated as the value lying 3 standard deviations above that of a zero sample (zero sample + 3 SD, repeatability, n = 21). \| \| 2 \| Special wash programming:  The use of special wash steps is mandatory when certain test combinations are run together on COBAS INTEGRA analyzers.  Introduction, Extra Wash Cycles for further instructions. Where required, special wash/carry-over evasion programming must be implemented prior to reporting results with this test \|   **Value**   \| Expected Values \| Reference Range \| \| Analytical Range \| Units \| \| --- \| --- \| --- \| --- \| --- \| \| Serum/plasma \| 2.8‑4.4 \| 0.3‑10.8 \| g/dL \| \| Newborns (0‑4 days): \| \| Children (4days‑14 years): \| 3.8-5.4 \| \| Children (14‑18 years): \| 3.2‑4.5 \| \| Adults (18‑60 years): \| 3.5‑5.2 \| |  |
| --- | --- | --- | --- | --- | --- | --- | --- | --- | --- | --- | --- | --- | --- | --- | --- | --- | --- | --- | --- | --- | --- | --- | --- |
|  |  |

Precision

| Performance Characteristics | Precision  Precision was determined using human samples and controls in an internal protocol with repeatability (n = 21) and intermediate precision (1 aliquot per run, 1 run per day, 10 days). The following results were obtained:   \| Repeatability \| Mean \| SD \| CV% \| Intermediate precision \| Mean \| SD \| CV% \| \| --- \| --- \| --- \| --- \| --- \| --- \| --- \| --- \| \| Serum low \| 25.5 \| 0.5 \| 1.8 \| Serum low \| 25.1 \| 0.3 \| 1.4 \| \| Serum high \| 64.0 \| 1.7 \| 2.6 \| Serum high \| 62.2 \| 1.5 \| 2.4 \| \| Precinorm protein \| 40.2 \| 1.0 \| 2.5 \| Precinorm protein \| 39.1 \| 1.1 \| 2.8 \| \| Precipath protein \| 61.4 \| 1.8 \| 2.9 \| Precipath protein \| 63.1 \| 1.8 \| 2.9 \|   Method comparison  Albumin values for human serum samples obtained on a COBAS INTEGRA 800 analyzer using the COBAS INTEGRA Tina‑quant Albumin Gen.2 reagent (y) was compared with those determined using the corresponding reagent on a cobas c 501 analyzer (x).  Cobas c 501 analyzer sample size (n)=80  Passing/bablok^15^ linear regression  y=0.903+0.875 y=0.875+2.336  T=0.945 r=0.995 |
| --- | --- | --- | --- | --- | --- | --- | --- | --- | --- | --- | --- | --- | --- | --- | --- | --- | --- | --- | --- | --- | --- | --- | --- | --- | --- | --- | --- | --- | --- | --- | --- | --- | --- | --- | --- | --- | --- | --- | --- | --- | --- |

**Subsidiary Document**

| Document unique ID | Document Name |
| --- | --- |
| NHIVRL/ALS/QPM/4.2-01 | Quality Policy Manual |

| Reference | - Roche Cobas Integra 400 plus& 501 Operator Manual - Roche Cobas Integra 400 & 501 plus Package Inserts - Tietz NW, editor. Text book of Clinical Chemistry.3^rd^ ed. Philadelphia: WB Saunders,2001 - Lothar Thomas: Clinical Laboratory Diagnostics, use and assessment of Clinical Laboratory Results 1^st^ ed.1998, |
| --- | --- |
