# Supplementary material for: The magnitude of undernutrition and associated factors among adult chronic kidney disease patients in selected hospitals of Addis Ababa, Ethiopia
Source: PLoS One. 2021 Jul 8;16(7):e0251730. doi: 10.1371/journal.pone.0251730 (PMC8266056; doi:10.1371/journal.pone.0251730)
Supplement: S2 File — (DOCX) [file pone.0251730.s002.docx]

## S2 File: Questionnaire English Version

**Code**

| Facility ________________________________  Data Collector’s Name _____________________ Supervisor’s name _______________________  Date _____________________ Date ____________________  Signature _________________ Signature _________________ | | | | | |
| --- | --- | --- | --- | --- | --- |
| **Part One: Socio Demographic And Economic Profile of Participant** | | | | | |
| 101 | Age | | __________ years |  | |
| 102 | Sex | | 1. Male 2. Female |  | |
| 103 | Place of Residence | | 1. Urban 2. Rural |  | |
| 104 | Marital Status | | 1. Married 2. Single 3. Divorced 4. Widowed |  | |
| 105 | Educational Level | | 1. No formal education 2. Primary education 3. Secondary education 4. Higher education |  | |
| 106 | Current Occupation | | 1. Farmer 2. Merchant 3. Daily laborer 4. Government employee 5. Non government employee 6. House wife 7. Other specify_______ |  | |
| 107 | What is your average monthly income (ETB)? | | _______________ ETB |  | |
| **Part Two: Clinical Profile of the participant** | | | | | |
| 201 | For How long have you been diagnosed with CKD | | ______ Years  ______ Months | Pt Card | |
| 202 | Cause of CKD | |  | Pt Card | |
| 203 | Stage of CKD/ Level of GFR/ | |  | Pt Card | |
| 204 | Current drug you are taking | |  | Pt card | |
| 205 | Family History of CKD | | 1. Yes 2. No |  | |
| 206 | Diabetes Miletus | | 1. Present 2. Absent | Pt card | |
| 207 | Hypertension | | 1. Present 2. Absent | Pt card | |
| 208 | Other Comorbidities | |  | Pt card | |
| **Part Three: Personal Behavior related Questions** | | | | | |
| 301 | Do you drink alcoholic beverages, like Teji, Tella, Beer, Arake, & the likes? | 1.Have never drank  2. I drink from time to time  4. I drink Daily | | |  |
| 302 | Do you smoke cigarettes? | 1. Have never smoke  2. I smoke from time to time  3. I smoke Daily | | |  |
| 303 | Do you do physical activity | 1. Yes 2. No | | |  |
| 304 | If yes for Q 303, how much time do you spend doing physical activities | 1.________ Hours per day  2._________ minutes per day  3.Don’t know  4.Not sure | | |  |
| **Part Four: Nutrition related Questions** | | | | | |
| 401 | How many regular meals do you eat in a typical day? | **________** | | |  |
| 402 | Do you get any nutrition counseling from health professionals? | 1. Yes 2. No | | |  |
| 403 | Do you participate in supplementary or therapeutic feeding program? | 1. Yes 2. No | | |  |
| 404 | Where are Your meals prepared | 1. At home 2. Out of home | | |  |

**Part Five: 24-Hour Dietary recall**

**Please describe the foods (meals and snacks) that you ate or drank yesterday during the day and night, whether at home or outside the home. Start with the first food or drink of the morning. Write down all foods and drinks mentioned. When composite dishes are mentioned, ask for the list of ingredients. When the respondent has finished, probe for meals and snacks not mentioned.**

| **Time/Meal** | **Place** | **Name of dish** | **Name of Ingredient** |
| --- | --- | --- | --- |
| **Breakfast** |  |  |  |
| **Snack** |  |  |  |
| **Lunch** |  |  |  |
| **Snack** |  |  |  |
| **Supper** |  |  |  |
| **Snack** |  |  |  |
|  | | | |

| **Part Six: Anthropometric Measurements** | | | | |
| --- | --- | --- | --- | --- |
|  |  | Measurement 1 | Measurement 2 | Measurement 3 |
| 601 | Height |  |  |  |
| 602 | Weight |  |  |  |
| **BMI** | | | | |
| **Part Seven: Results of Laboratory Measurements** | | | | |
| **701** | Serum Albumin |  |  |  |
|  |  |  |  |  |
|  |  |  |  |  |
