# Supplementary material for: The magnitude of undernutrition and associated factors among adult chronic kidney disease patients in selected hospitals of Addis Ababa, Ethiopia
Source: PLoS One. 2021 Jul 8;16(7):e0251730. doi: 10.1371/journal.pone.0251730 (PMC8266056; doi:10.1371/journal.pone.0251730)
Supplement: S3 File — (DOCX) [file pone.0251730.s003.docx]

## S3 File: Amharic Version of Questionnaire

## መጠይቅ

**መ ለያ ቁጥር**

| **ድርጅት ________________________________**  **የመረጃሰብሳቢውስም _____________________ የተቆጣጣሪውስም_______________________**  **ቀን_____________________ ቀን ____________________**  **ፊርማ _________________ ፊርማ_________________** | | | | | |
| --- | --- | --- | --- | --- | --- |
| ክፍልአንድ፡የተሳታፊውመሰረታዊመረጃ | | | | | |
| 101 | እድሜ | | __________ ዓመት |  | |
| 102 | ፆታ | | 1.ወንድ  2.ሴት |  | |
| 103 | የመኖሪያአድራሻ | | 1.ከተማ  2.ገጠር |  | |
| 104 | የጋብቻሁኔታ | | 1.ያገባ  2.ያላገባ  3.የፈታ/ች  4.አግብቶየሞተበት/ባት |  | |
| 105 | የትምህርትደረጃ | | 1.ያልተማረ  2.አንደኛደረጃ  3.ሁለተኛደረጃ  4.ከፍተኛትምህርት |  | |
| 106 | አሁንየሚሰሩትየስራአይነት | | 1.ገበሬ  2.ነጋዴ  3.የጉልበትሰራተኛ  4.የመንግስትሰራተኛ  5.የግልሰራተኛ  6.የቤትእመቤት  7.ሌላካለይገለፅ ----------------- |  | |
| 107 | አማካይ ወርሃዊገቢዎ (በኢትዮጵያብር) | | _______________ ብር |  | |
| **ክፍልሁለት: የተሳታፊው የጤና ሁኔታ** | | | | | |
| 201 | በሽታውእንዳለብዎካወቁስንትጊዜሆኖት | | --------- ዓመት | ከታማሚውካርድ | |
| 202 | የበሽታውመንስኤ | |  | ከታማሚውካርድ | |
| 203 | የበሽታውደረጃ | |  | ከታማሚውካርድ | |
| 204 | አሁንእየወሰዱያለውመድሃኒት | |  | ከታማሚውካርድ | |
| 205 | በቤተሰብዎውስጥተመሳሳይበሽታያለበትሰውአለ | | 1.አዎ  2.አይደለም |  | |
| 206 | በሃኪም የተረጋገጠ የስኳርበሽታአለብዎት | | 1.አዎ  2.አይደለም | ከታማሚውካርድ | |
| 207 | የደም ግፊት በሽታ አለብዎት | | 1.አዎ  2.አይደለም | ከታማሚውካርድ | |
| 208 | ሌላ ተጓዳኝ በሽታ ካለብዎት ይገለፅ | | ------------- | ከታማሚውካርድ | |
| **ክፍል ሶስት፡ ከተሳታፊው የግል ባህሪ ጋር የተያያዙ ጥያቄዎች** | | | | | |
| 301 | የአልኮል መጠጦችን እንደ ጠጅ ጠላ ቢራ አለቄ እና የመሳሰሉትን ጠጥተው ያውቃሉ | 1.ጠጥቼ አላውቅም  2. አልፎ አልፎ እጠጣለሁ  3.በየቀኑ እጠጣለሁ | | |  |
| 302 | ሲጋራያጨሳሉ | 1. አጭሼ አላውቅም  2. አልፎ አልፎ አጨሳለሁ  3.በየቀኑ አጨሳለሁ | | |  |
| 303 | የአካል ብቃት እንቅስቃሴ ያደርጋሉ | 1.አዎ  2.አላደርግም | | | አዎከሆነመልስዎወደጥያቄቁጥር 304 ይሂዱ |
| 304 | ምን ያህል ጊዜ የአካል ብቃት እንቅስቃሴ በመስራት ያጠፋሉ | ________ ሰዓት/በቀን  _________ ደቂቃ/በቀን  አላውቅም  እርግጠኛ አይደለሁም | | |  |
| **ክፍል አራት : የስነ አመጋገብ ጥያቄዎች** | | | | | |
| 401 | በቀን ውስጥ ስንት ጊዜ ይመገባሉ ? | **__________** | | |  |
| 402 | ስለ አመጋገብ ምክር ከጤና ባለሞያዎች አግኝተው ያውቃሉ | 1.አዎ  2.አይ | | |  |
| 403 | ከህመም ጋር በተያያዘ ተጨማሪ ምግብ ከጤና ተቋም ተሰጥቶት ያውቃል | 1.አዎ  2.አይ | | |  |
| 404 | ምግብዎ ብዙ ጊዜ የት ነው የሚዘጋጀው | 1. ቤት ውስጥ  2.ከቤት ውጪ | | |  |
|  |  |  | | |  |
|  |  |  | | |  |

**ክፍል አምስት፡ ትላንት ከቁርስ እስከ እራት የተመገቡት ምግብ**

እባኮትን ትላንት ከቁርስ እስከ እራት ሰዓት ቤት ውስጥ ወይንም ከቤትዎ ውጪ የተመገቡትን ምግብ ወይንም የጠጡትን መጠጥ ይግለፁ፡፡ ጠዋት መጀመሪያ በተመገቡት ወይንም ከጠጡት ይጀምሩ፡፡ የተጠቀሱትን ሁሉንም ምግቦች ይዘርዝሩ፡፡ ሙሉ የሆኑ ምግቦች ከተጠቀሱ የተሰሩበትን ንጥረ ነገር ይዘርዝሩ፡፡ መላሹ መልሶ ሲጨርስ እርስዎ ያልተሟሉ ምግቦች ካሉ ይጠይቁ፡፡

| **ጊዜ/ምግብ** | **ቦታ** | | **የምግቡ አይነት** | | | **ምግቡ የተሰራበት ንጥረ ነገር** | | |
| --- | --- | --- | --- | --- | --- | --- | --- | --- |
| **ቁርስ** |  | |  | | |  | | |
| **መክሰስ** |  | |  | | |  | | |
| **ምሳ** |  | |  | | |  | | |
| **መክሰስ** |  | |  | | |  | | |
| **እራት** |  | |  | | |  | | |
| **መክሰስ** |  | |  | | |  | | |
| **ክፍል ስድስት፡ የሰውነት ልኬት መግለጫ** | | | | | | | |  |
|  | |  | | ልኬት አንድ | ልኬት ሁለት | | ልኬት ሶስት |  |
| 601 | | ቁመት | |  |  | |  |  |
| 602 | | ክብደት | |  |  | |  |  |
| **BMI** | | | | | | | |  |
| **ክፍል ሰባት: የደም ናሙና የላብራቶሪ ውጤት** | | | | | | | |  |
| **701** | | Serum Albumin | |  |  | |  |  |
