# Supplementary material for: The magnitude of undernutrition and associated factors among adult chronic kidney disease patients in selected hospitals of Addis Ababa, Ethiopia
Source: PLoS One. 2021 Jul 8;16(7):e0251730. doi: 10.1371/journal.pone.0251730 (PMC8266056; doi:10.1371/journal.pone.0251730)
Supplement: Attachment — Submitted filename: Response to reviewers.docx [file pone.0251730.s004.docx]

**Response to reviewers**

**General note for academic editor regarding the overall progress**

First of all, we would like to acknowledge the academic editor for giving us adequate time to revise and address all the concerns of the reviewers and journal requirements. Following, we the authors of this manuscript have been working extensively since we have been notified with the academic editor and expert reviewer’s reports of the manuscript giving a due attention for all the concerns raised by the academic editor and expert reviewers to be well addressed.

The presentations of the results of the study have been critically worked and made to be in a logical and coherent sequence, study limitations and interpretations are re-written and sufficiently and clearly elaborated. The whole manuscript file has almost been re-written correcting all the syntax and grammatical errors. We hope the academic editor and the expert reviewers get it as much improved manuscript. Thank you so much!

A. **Point by point response letter to academic editor**
1. We have checked again our manuscript for fulfillment of PLOS ONEs style requirements including the naming of files and it has been written accordingly. Thank you!!
2. We have amended the abstract and placed the abstract after the title page as per the recommendation. Thank you!!
3. We placed the ethics approval statement at the end of methods section only and removed it from the prior section of the manuscript. Thank you in advance!!!
4. As per your recommendation, we inserted caption for figure 2 .Thank you in advance!!!
B. **Point by point response letter to reviewer one**

First of all we would like to express our gratefulness for the reviewer for appropriately recognizing the topic as one of the important area of research and for being interested on the topic. Following our acknowledgement, the reviewers concerns are point by point addressed in the following bullets

- The reviewer’s comment regarding the papers being unnecessarily long is well accepted. We make the current revised version clear and focused only to the study objectives. All the other out of the scope of the study which made the paper unnecessarily long have been removed and the study objectives related contents are further and adequately elaborated. We hope the reviewer will get the revised version clear and to the point of the study objectives. Thank you so much!
- The reviewer’s comment regarding the language errors are also the right concerns and we have re-written each and every statement throughout the manuscript correcting all the grammatical and punctuation errors consulting English language experts in Wolaita Sodo University. We hope the reviewer will get the revised version is clear and improved. Thank you!
- The reviewer’s comments regarding methods, discussion and tables of the previous manuscript require significant revision is well accepted. In the current revised version, we made all the methods followed during the study are well and clearly explained, in the discussion section the important finding are clearly discussed, tables and figures are well revised. Thank you!
- The reviewer’s comments about the discussion sections lack of sufficient circumspection both in the interpretation and relationship with the extant literature are right. In the recent revised version we have appropriately re-written the whole discussion section and we made the interpretations clear and discussed the findings with relevant and up to date results in the extant literature. Thank you!
- As overall, the recent revised manuscript file is adequately renovated version and we hope the reviewer will get the manuscript as much improved. Thank you so much in advance!

C. **Point by point response letter to reviewer two**

- Before all, we would also like to express our thankfulness of reviewer two for carefully and critically reviewing this manuscript sacrificing crucial time and effort for maintaining scientific integrity of the manuscript and to be considered for publication meeting the scientific and journal requirements. Following our gratefulness of the reviewer, we have addressed all the reviewers concerns point by point as follows:

**Point by point response to major comments of reviewer two**

1. The reviewers concerns about revision of the language and formatting reference list is well accepted. Accordingly, we have thoroughly read the whole manuscript file’s each and every statement for any grammatical errors and corrected throughout the document and we have formatted the reference list as it appears in the manuscript document sequentially. Thank you!
2. The reviewers comments regarding the structure of the introduction section of the previous version has been accepted and we re-structured the introduction section of this revised manuscript as per the reviewers recommendation in to three parts “what have been known in the existing body of knowledge”, “what is lacking in the current literature” and “the hypothesis of the this particular study”. Thank you so much!
3. The reviewer’s comment to explicitly define “undernutrition” in methods section is accepted and as per the reviewers recommendation we have explicitly defined undernutrition and other relevant concepts in the methods section of the manuscript. Thank you!
4. The reviewer’s comments regarding the some important variables lack of unit of measurement (age in years and currency) and mentioning Table 3 before Table 2 have been accepted. We have included the units of measurements for age and currency and all the tables are sequentially placed in the manuscript file. Thank you!
5. The authors would like to thank the reviewer for the best insight regarding incomparable referencing of the characteristics of CKD patients. Reference number 6 has been removed from the text and reference list, because the study was on CKD patients who were on hemodialysis which is not in the scope of this study and the concept held in reference number 7 has been elaborated further because it is a matched reference with the current study. Thank you!
6. The reviewer’s comments about unsatisfactorily addressing of the limitations of the study in the previous version are right and well accepted. In the current revised version, we have appropriately and satisfactorily addressed the limitations of the study including all the possible limitations of this particular study. We hope the reviewer will get the concerns are well addressed. Thank you in advance!

**Point by point response to minor comments of reviewer two**

1. The reviewer’s comment regarding the number of participants to be included in the abstract results part is right and it has been included. Thank you!
2. According to the reviewers comment, we noticed the phrase “diabetic glomerulonephritis” was unnecessary and irrelevant to this particular study and it has been removed from the manuscript file. Instead, we have operationalized the term “glomerulonephritis” which is an important variable for the study and included in operational definition section of the main manuscript file. Thank you!
3. The reviewers comments regarding the comparison of the “prevalence of undernutrition among chronic kidney disease patients” in the abstract conclusion section is accepted. As per the suggestion of the reviewer in the current revised version the “prevalence of undernutrition among chronic kidney disease patients” has been described relative to its public health significance when compared to its prevalence in the Sub-Saharan Africa region. Thank you!
4. The reviewer’s comment regarding the words which are unnecessarily terrifying has been accepted and replaced with appropriate ones. Thank you!

Thank you!
