# Supplementary material for: The magnitude of undernutrition and associated factors among adult chronic kidney disease patients in selected hospitals of Addis Ababa, Ethiopia
Source: PLoS One. 2021 Jul 8;16(7):e0251730. doi: 10.1371/journal.pone.0251730 (PMC8266056; doi:10.1371/journal.pone.0251730)
Supplement: Attachment — Submitted filename: Response to reviewer.docx [file pone.0251730.s005.docx]

**General note to academic editor**

First of all, we would like to express gratitude for the academic editor for giving us sufficient time to address all concerns of the reviewer. Following, our acknowledgment we have been working extensively since we have been notified to address the concerns of the expert reviewer and explained point by point in the following bullets.

**Point by point response for reviewer 2**

- Before all, we would like to express our thankfulness of reviewer two for carefully and critically reviewing revised manuscript. We found the comments and recommendations of the reviewer are very important inputs to maintain standards of the journal requirements for publication.

1. **Point by point response to vocabulary and grammar errors for reviewer**

- The reviewer comment regarding vocabulary and grammar errors are the right concerns. We have incorporated the recommendations of the reviewer and also checked the whole manuscript for similar errors and corrected accordingly. We hope the reviewer can access all the revisions in the clean copy of the manuscript and also from the revised manuscript with track changes. Thank you in so much!

2. **Point by point response to minor comments of reviewer 2**

- The reviewer comment regarding to remove 160 and leave 43.1% for the prevalence is acceptable and we have corrected accordingly. Thank you!
- The concern of the reviewer about the definition of glomerulonephritis is right. However, we defined it as it appears in the studied hospitals diagnostic criteria. Thank you!

3. **Point by point response to major comments of reviewer 2**

- The reviewer concern regarding the cut-point off hypoalbuminemia is appropriate and right. However, when we collect the information for the study, we have raised the concern with the clinicians on follow up care for the CKD patients. The normal serum albumin for general population ranges from 3.5g/dl- 5.2g/dl. The minimum cut- off value of 3.8g/dl was being used as a lower limit to consider hypoalbuminemia in the studied hospitals because CKD patients are vulnerable group for nutritional anemia. To avoid any delays in nutritional interventions for the CKD patients, the lower cut-off limit has been raised from 3.5g/dl to 3.8g/dl. This is why the studied hospitals are using 3.8g/dl as lower limit to consider hypoalbuminemia and we have used the same lower limit for the study. Thank you so much!
- According to the reviewer suggestion we have removed the comparison of the findings from the conclusion section and associated factors with the dependent variable has been stated in the current revised version of the manuscript. Thank you!
- The reviewer comment regarding explicit definition of the undernutrition in the abstract and the method sections is appropriate and acceptable. We have explicitly defined undernutrition as BMI<18.5 in both sections. Thank you!
- The reviewer comment regarding the strength of the study section is right. We have revised the section in the recent revised version and made clear. Thank you!
